# Association of *NAT2* promoter hypermethylation with susceptibility to hepatotoxicity due to antituberculosis drugs and biomarker potential

**DOI:** 10.1038/s41598-025-95050-6

**Published:** 2025-03-25

**Authors:** Jiraphun Jittikoon, Wacharapol Saengsiwaritt, Noppadol Chanhom, Usa Chaikledkaew, Sukanya Wattanapokayakit, Surakameth Mahasirimongkol, Wanvisa Udomsinprasert

**Affiliations:** 1 Department of Biochemistry, Faculty of Pharmacy, Mahidol University, 447 Sri-Ayudthaya Road, Rajathevi, Bangkok, 10400 Thailand; 2 Social and Administrative Pharmacy Division, Department of Pharmacy, Faculty of Pharmacy, Mahidol University, Bangkok, 10400 Thailand; 3 Mahidol University Health Technology Assessment (MUHTA) Graduate Program, Mahidol University, Bangkok, 10400 Thailand; 4 Division of Genomic Medicine and Innovation Support, Department of Medical Sciences, Genomic Medicine Centre, Ministry of Public Health, Nonthaburi, 11000 Thailand

**Keywords:** *N*-Acetyltransferase 2 (*NAT2*), Promoter methylation, Anti-tuberculosis drug-induced liver injury, Tuberculosis, Biomarker, Genetics, Biomarkers, Diseases

## Abstract

This study aimed to determine whether promoter methylation of *N*-acetyltransferase 2 (*NAT2*), a metabolic enzyme responsible for drug metabolism and detoxification, was correlated with clinical parameters indicating anti-tuberculosis drug-induced liver injury (ATDILI) in tuberculosis patients and might emerge as an ATDILI biomarker. *NAT2* promoter methylation in blood leukocyte of 102 tuberculosis patients (49 ATDILI cases and 53 non-ATDILI cases) and 100 healthy controls were quantified using quantitative real-time methylation-specific polymerase chain reaction. Compared to healthy volunteers, tuberculosis patients had significantly reduced *NAT2* demethylation index. Compared with non-ATDILI patients, *NAT2* demethylation index was significantly decreased in ATDILI patients. An independent association was found between lower *NAT2* demethylation index and increased susceptibility to ATDILI. *NAT2* demethylation index quantified after starting treatment within 1–7 days was negatively correlated with serum aminotransferases measured within 8–60 days of treatment. ROC curve analysis uncovered that *NAT2* demethylation index was found to be a more sensitive and specific biomarker for ATDILI when compared to serum aminotransferases measured following treatment initiation within 1–7 days. Kaplan–Meier analysis unveiled a notable association between lower *NAT2* demethylation index and a higher incidence of ATDILI in tuberculosis patients, as confirmed by Cox regression analysis while accounting for confounding variables. A reduction in *NAT2* demethylation index could reflect ATDILI progression and potentially be used as a new, specific biomarker for ATDILI.

**Supplementary Information:**

The online version contains supplementary material available at 10.1038/s41598-025-95050-6.

## Introduction

Tuberculosis is a prevalent and serious opportunistic infection caused by the bacterium *Mycobacterium tuberculosis*, and it is increasingly recognized as a significant global health concern^1^. The administration of rifampicin, isoniazid, pyrazinamide, and ethambutol as the initial treatment for tuberculosis has demonstrated efficacy in reducing the rising prevalence of the disease. However, it is worth noting that this combination therapy often results in the manifestation of severe adverse events^2^. Hepatotoxicity due to anti-tuberculosis drugs, also referred to as anti-tuberculosis drug-induced liver injury (ATDILI), is a commonly encountered and significant adverse effect observed in the course of tuberculosis treatment. This condition has been observed to adversely affect medication adherence in patients with tuberculosis, leading to treatment failure^3,4^. In severe cases, ATDILI has the potential to lead to acute liver failure, necessitating liver transplantation for prolonged survival in certain individuals. Consequently, this presents a notable obstacle in the timely management of tuberculosis progression. Within this particular context, the prompt and precise identification of ATDILI holds significant importance in enhancing management and control of tuberculosis. The current identification of ATDILI involves the assessment of liver function biomarkers, with a particular emphasis on alanine aminotransaminase (ALT), which is widely recognized as the benchmark for evaluating liver damage. Nevertheless, there have been reports indicating that increased levels of ALT are not exclusive to DILI^5^. This is due to the fact that metabolic disturbances can lead to false positive results^6,7^. Given the aforementioned challenges, it is imperative to ascertain precise and mechanistic biomarkers of ATDILI. This endeavor holds the potential to enhance the efficacy of tuberculosis treatment. In relation to this issue, acquiring a more comprehensive understanding of the underlying causes linked to ATDILI would provide valuable insights into identifying mechanistic biomarkers of ATDILI.

Recent advancements in genome-wide studies have provided evidence suggesting that the presence of genetic and epigenetic diversity can contribute to variations in individuals’ responses to drugs and their susceptibility to toxicity^8^. It is important to understand how genetic variations, in conjunction with environmental factors, can impact the development and progression of DILI^9^. Epigenetic mechanisms refer to heritable changes in gene expression that occur without alterations in the DNA sequence. These regulatory mechanisms play a crucial role in controlling gene activity and include histone modifications, non-coding RNAs, and DNA methylation^10^. Histone modifications, such as acetylation, methylation, and phosphorylation, influence chromatin structure and gene accessibility. Additionally, non-coding RNAs, including microRNAs and long non-coding RNAs, regulate gene expression post-transcriptionally^11^. DNA methylation, the most well-characterized epigenetic modification, involves the addition of a methyl group to cytosine residues in CpG dinucleotides by DNA methyltransferases, typically leading to transcriptional repression^12^. This mechanism is crucial in regulating gene expression without altering the DNA sequence, leading to reduced transcriptional activity or gene repression^13,14^. Over the past years, there has been a growing body of research aiming to establish a connection between epigenetic marks, specifically DNA methylation within the promoter regions of genes encoding metabolic enzymes, and ATDILI development^15–18^. Of various drug-metabolizing enzymes, *N*-acetyltransferase 2 (NAT2), an important Phase II metabolic enzyme intricately engaged in drug metabolism and detoxification^19^, has garnered growing attention as a potential molecule associated with ATDILI development^20^. On the basis of its biological function, the investigation into *NAT2* promoter methylation holds considerable importance in understanding ATDILI development and may pave the way for the identification of a new, specific biomarker for ATDILI.

Although the significant involvement of *NAT2* promoter methylation in ATDILI has been previously studied in the Mongolian population^21^, its relationship with ATDILI in the Thai population has not been explored. Accordingly, this study aimed to investigate the potential association between *NAT2* promoter methylation clinical parameters indicating ATDILI and to assess whether *NAT2* promoter methylation could serve as a specific biomarker of ATDILI in tuberculosis patients.

## Materials and methods

The study protocol received approval from the Institutional Review Board of the Faculty of Dentistry/Faculty of Pharmacy, Mahidol University (MU-DT/PY-IRB 2023/025.2003) and was conducted in accordance with the principles outlined in the Declaration of Helsinki. All participants were provided with comprehensive information regarding the study protocols and procedures. Prior to their enrollment in this study, all subjects provided written informed consent.

### Study participants

This study was conducted across multiple centers and employed a case–control design. The study population consisted of 102 individuals diagnosed with tuberculosis based on clinical blood tests, a simple skin test, and histological findings and 100 healthy controls who participated in an annual health examination at Chiang Rai Prachanukroh Hospital. Healthy controls were selected using convenience sampling and were required to have no clinical signs or symptoms of tuberculosis, autoimmune diseases, or liver diseases, as confirmed by medical records and consultations. Short-course anti-tuberculosis drugs were administered to all tuberculosis patients from the 10 designated hospitals, namely Bangplama Hospital in Suphan Buri, The Central Chest Disease Institute in Nonthaburi, Chiang Rai Prachanukroh Hospital in Chiang Rai, Hatyai Hospital in Songkla, Maesot Hospital in Tak, Nopparat Rajathanee Hospital in Bangkok, Buddhachinaraj Hospital in Phitsanulok, Ramathibodi Hospital in Bangkok, Rayong Hospital in Rayong, and Thai Mueang Chaipat Hospital in Phang-nga. In accordance with the World Health Organization guidelines^22,23^, the drug regimen consisted of rifampicin (8–12 mg/kg once-daily dosing), isoniazid (4–8 mg/kg once-daily dosing), pyrazinamide (20–30 mg/kg once-daily dosing), and ethambutol (15–20 mg/kg once-daily dosing) for the initial 2 months, followed by rifampicin (8–12 mg/kg once-daily dosing) and isoniazid (4–8 mg/kg once-daily dosing) for the subsequent 4 months. Regarding hepatotoxicity, tuberculosis patients enrolled in the study were divided into two groups based on their blood levels of liver function tests: those with ATDILI (*n* = 49) and those without ATDILI (*n* = 53). According to the clinical practice guidelines for tuberculosis treatment in Thailand^24^, ATDILI cases were considered to meet the criteria if they fulfilled at least one of the following conditions: (1) Elevated levels of aspartate aminotransferase (AST) and ALT that exceed five times the upper limit of normal (ULN). (2) Elevated levels of AST and/or ALT that exceed three times the ULN, accompanied by at least one symptom of hepatitis such as anorexia, fatigue, nausea, vomiting, jaundice, liver enlargement, and/or dark urine. (3) Elevated levels of AST and/or ALT, with or without symptoms of hepatitis, along with an increase in total bilirubin that exceeds three times the ULN. This study excluded individuals with additional hepatic conditions, such as viral hepatitis or chronic liver dysfunction, those who displayed abnormal liver function tests at the beginning of the study, and those who were administered other hepatotoxic medications.

### Sample size calculation

The statistical power of our study was calculated using G*Power software employing a case–control design with a binary outcome (ATDILI vs. non-ATDILI). Drawing on prior studies and preliminary data^15,17^, we estimated an expected effect size (Cohen’s d) of 0.5, a two-sided significance level (α) of 0.05, and a desired statistical power of 80% (β = 0.20). Using these parameters, the required sample size was estimated to be approximately 100 participants per group to achieve optimal power for detecting a statistically significant association. However, due to practical constraints, our study included 49 ATDILI cases and 53 controls, resulting in a power of approximately 70–75%. While this is slightly below the ideal threshold, it remains sufficient for an exploratory analysis.

### Collection of blood samples and assessment of clinical parameters

Peripheral blood samples were collected from healthy controls and tuberculosis patients who had received treatment with anti-tuberculosis drugs. Tuberculosis patients were closely monitored throughout treatment for signs of ATDILI. Clinical and biochemical evaluations, including liver function tests, were conducted at baseline and during designated follow-up appointments. The initial blood sample was collected between 1 and 7 days after the commencement of treatment to evaluate pre-existing conditions and provide a reference for subsequent analyses. These baseline samples were also used for DNA extraction to assess *NAT2* promoter methylation status. A second blood sample was obtained between 8 and 60 days post-initiation of treatment, primarily for biochemical analysis. This sample facilitated the evaluation of any potential early liver injury or alterations in biomarkers throughout the treatment period. The follow-up was conducted through systematic clinical monitoring, during which patients were observed for any indications of liver dysfunction, and their liver function tests were meticulously evaluated.

Liver function tests were routinely assessed using an automated analyzer to measure crucial biochemical parameters, including aspartate aminotransferase (AST), alanine aminotransferase (ALT), alkaline phosphatase (ALP), total bilirubin, and direct bilirubin.

### DNA extraction and bisulfite treatment

Genomic DNA from peripheral blood lymphocytes was purified using the QIAamp DNA Blood Mini Kit (Qiagen, CA, USA), following the manufacturer’s protocol. Extracted DNA was quantitated using a NanoDrop 2000 spectrophotometer. The DNA (50 ng) was then treated with sodium bisulfite using the EZ DNA Methylation Gold Kit (Zymo Research, Orange, CA, USA), following the manufacturer’s protocol.

### Quantitative real-time methylation-specific polymerase chain reaction (qMSP)

**Fig. 1:**
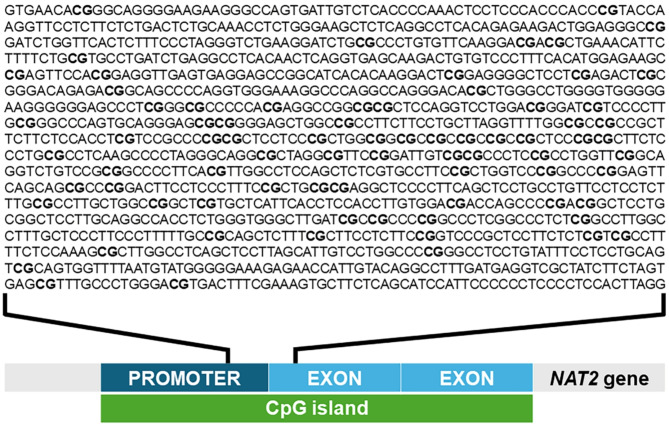
Location of methylation sites assessed within promoter region of *NAT2* gene**.**

The measurement of *NAT2* promoter methylation was conducted using qMSP, which has been previously employed to quantify methylation levels, as previously detailed^25^. Briefly, the bisulfite-treated DNA underwent amplification through real-time PCR using primers specifically designed by MethPrimer (https://www.urogene.org/cgi-bin/methprimer/methprimer.cgi). As displayed in Fig. 1, the primers were designed for the methylation-favorite site of the *NAT2* promoter sequences, which were predicted using an online prediction website. There are two types of primers available for different sequences. One type was designed for fully methylated sequences and can identify unconverted cytosine after bisulfite treatment. The other type of primer was used for fully unmethylated sequences and can bind to uracil, which is converted from cytosine. The primer sequences were as follows: m*NAT2* forward 5′-TTCGGTTTCGGAGTTTAGTAGC-3′, m*NAT2* reverse 5′-GTCCACAAAATAAAAATAAATAAACACG-3′, u*NAT2* forward 5′-GGTTTTGGTTTTGGAGTTTAGTAGT-3′, and u*NAT2* reverse 5′-CATCCACAAAATAAAAATAAATAAACACA-3′. A real-time PCR experiment was conducted using the StepOnePlus™ real-time PCR system (Applied Biosystems, Foster City, CA, USA) with an initial step at 95 °C for 15 min, followed by 40 cycles of temperature 94 °C for 15 s, 58 °C for 30 s, and 72 °C for 50 s. The PCR mixture included bisulfite-converted DNA, 2× SYBR Green PCR Master Mix (Biotechrabbit GmbH, Hennigsdorf, Germany), and both forward and reverse primers. All procedures were performed in duplicate to ensure accuracy and consistency. The results were then averaged after analysis. The relative demethylated DNA was determined using the following equation: demethylation index = 2^(methylated cycle number) − (demethylated cycle number)^, as previously described^26^.

### Statistical analysis

The statistical analyses were conducted using the statistical package for social sciences version 26.0 (SPSS, Inc., Chicago, IL, USA). Demographic and clinical characteristics between groups were assessed using statistical tests appropriate for each variable type. Categorical variables were compared using the Chi-square (χ^2^) test, reported as percentages. Continuous variables were compared using the Mann Whitney U test, reported as median with interquartile ranges (IQR). Among groups, continuous variables described as median with IQR were compared using the Kruskal–Wallis H test. The association between two variables was analyzed using Pearson’s correlation. A multivariate regression analysis was conducted to control the effect of confounding factors such as age, sex, body mass index (BMI), drinking status, smoking status, and the timing of blood collection on the outcome of interest. The diagnostic accuracy of biomarkers for ATDILI was assessed by constructing a receiver operating characteristic (ROC) curve. For assessing the potential of *NAT2* promoter methylation as a prognostic marker for ATDILI, Kaplan–Meier curves were generated for all patients categorized into low or high methylation levels of *NAT2*. These categories were determined using the optimal cut-off point obtained from ROC curve analysis. All analyses were considered statistically significant if the *P* value was less than 0.05.

## Results

### Demographic and clinical characteristics of study subjects

Baseline demographic and clinical characteristics of tuberculosis patients with and without ATDILI are detailed in Table 1. After 1–7 days of starting treatment, no significant differences in age, gender ratio, BMI, and liver function tests including ALT, AST, total bilirubin, and direct bilirubin between tuberculosis patients with and without ATDILI were observed. As expected, tuberculosis patients who experienced ATDILI displayed significantly elevated levels of ALT, AST, total bilirubin, and direct bilirubin compared to those without ATDILI within 8–60 days of initiating treatment (*P* < 0.001, *P* < 0.001, *P* < 0.001, *P* = 0.027, respectively). In the comparison of baseline characteristics between tuberculosis patients and healthy controls, there were no significant differences observed in median age, gender ratio, median BMI, and clinical parameters consisting of ALT, AST, total bilirubin, and direct bilirubin (Supplementary Table S1).

**Table 1:** Baseline and clinical characteristics of tuberculosis patients with and without ATDILI after commencement of anti-tuberculosis treatment.

Variables	Tuberculosis patients		*P* value^a^
	ATDILI	Non-ATDILI	
Number	49	53	N/A
Age (years)	48.00 (39.50, 65.00)	49.00 (33.00, 60.50)	0.094
Gender (F/M)	21 (42.90%)/28 (57.10%)	16 (30.20%)/37 (69.80%)	0.220
BMI (kg/m^2^)	18.94 (16.03, 22.10)	20.06 (17.34, 21.17)	0.383
Drinking status			
Never/ever	32 (65.31%)/17 (34.69%)	32 (60.38%)/21 (36.92%)	0.341
Smoking status			
Never/ever	32 (65.31%)/17 (34.69%)	32 (60.38%)/21 (36.92%)	0.341
Biochemical parameters			
Within 1–7 days of treatment			
ALT (IU/L)	25.00 (12.00, 35.00)	27.00 (23.00, 33.00)	0.228
AST (IU/L)	15.00 (15.00, 28.00)	25.00 (22.25, 32.50)	0.065
Total bilirubin (mg/dL)	0.60 (0.48, 0.76)	0.40 (0.30, 0.65)	0.062
Direct bilirubin (mg/dL)	0.28 (0.11, 0.40)	0.13 (0.10, 0.23)	0.172
Within 8–60 days of treatment			
ALT (IU/L)	126.00 (84.50, 163.50)	20.00 (16.00, 30.00)	**< 0.001**
AST (IU/L)	179.00 (111.50, 245.00)	25.00 (20.00, 34.00)	**< 0.001**
Total bilirubin (mg/dL)	1.51 (0.60, 2.50)	0.50 (0.38, 0.80)	**< 0.001**
Direct bilirubin (mg/dL)	0.75 (0.28, 1.60)	0.26 (0.13, 0.40)	**0.027**

Data are represented as either median with interquartile range (IQR) for continuous variables or percentages for categorical variables.

*P* values marked with bold indicate statistically significant differences between the groups.

*ALP* alkaline phosphatase, *ALT* alanine aminotransferase, *AST* aspartate aminotransferase, *ATDILI* anti-tuberculosis drug-induced liver injury, *BMI* body mass index, *F* female, *N/A* not available, *M* male.

^a^Comparisons in baseline demographic and clinical parameters between tuberculosis patients with ATDILI and those with non-ATDILI.

### *NAT2* hypermethylation in tuberculosis patients with ATDILI

**Fig. 2:**
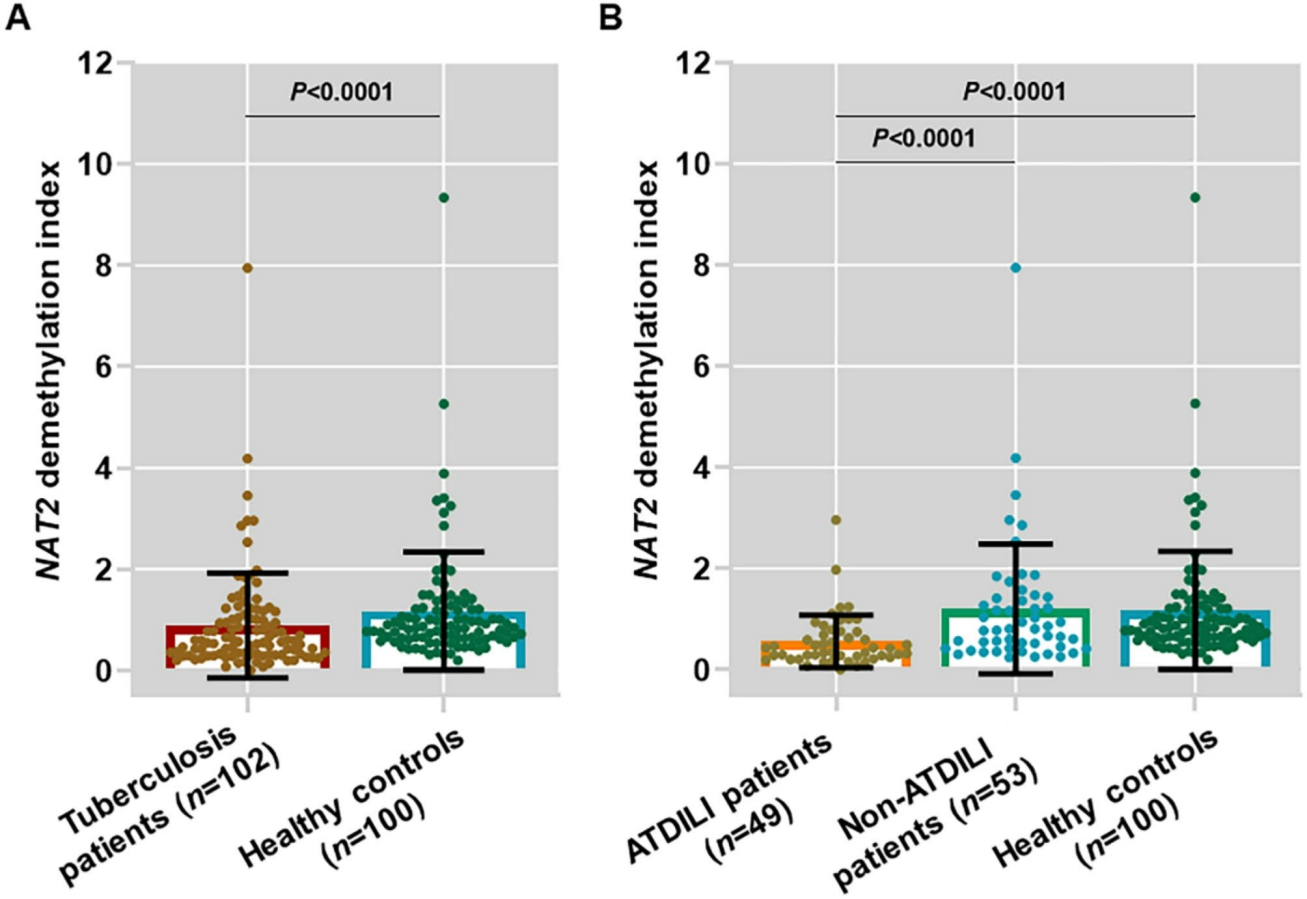
*NAT2* demethylation index of tuberculosis patients with and without ATDILI as well as healthy controls. (**A**) Comparison of *NAT2* demethylation index between tuberculosis patients and healthy controls. (**B**) Comparison of *NAT2* demethylation index between tuberculosis patients with ATDILI and without ATDILI. *P* values were derived from Mann Whitney U test (**A**) and Kruskal–Wallis H test.

Compared to healthy controls, tuberculosis patients had significantly reduced *NAT2* demethylation index, indicating *NAT2* hypermethylation in tuberculosis patients (*P* < 0.0001) (Fig. 2A). In tuberculosis patients with ATDILI, *NAT2* demethylation index was found to be significantly decreased when compared to healthy controls (*P* < 0.0001) (Fig. 2B). In comparison to tuberculosis patients without ATDILI, patients with ATDILI had a significant decrease in *NAT2* demethylation index (*P* < 0.0001) (Fig. 2B). These findings indicate *NAT2* hypermethylation in tuberculosis patients, particularly those with ATDILI.

### *NAT2* hypermethylation as an independent determinant of ATDILI

Considering potential confounding factors such as age, gender, BMI, drinking status, smoking status, and the timing of blood collection, it was necessary to determine whether *NAT2* promoter methylation was an independent risk factor of ATDILI. After accounting for confounding factors, it was found that *NAT2* demethylation index in tuberculosis patients with ATDILI remained significantly lower than that in those without ATDILI (odds ratio, OR 8.88; 95% CI 1.30, 60.87; *P* = 0.026). Using the cut-off value accurately discriminating ATDILI patients from those without ATDILI, as determined through ROC curve analysis, *NAT2* demethylation index was divided into two groups: *NAT2* hypermethylation (*NAT2* demethylation index < 0.52, *n* = 46) and *NAT2* hypomethylation (*NAT2* demethylation index ≥ 0.52, *n* = 56). After accounting for the factors mentioned earlier, a multivariate logistic regression analysis revealed a strong association between *NAT2* hypermethylation and a significantly increased risk of ATDILI in tuberculosis patients. The risk was found to be 4.62 times greater in tuberculosis patients with *NAT2* hypermethylation than those with *NAT2* hypomethylation (OR 4.62; 95% CI 1.12, 19.05; *P* = 0.034) (Table 2).

**Table 2:** Multivariate linear regression analysis of associations between *NAT2* demethylation index measured within 1–7 days of treatment and clinical parameters assessed within either 1–7 days or 8–60 days of treatment initiation in tuberculosis patients.

Variables	Tuberculosis patients with and without ATDILI	
	OR (95%CI)	*P* value^a^
Overall	8.88 (1.30, 60.87)	**0.026**
*NAT2* demethylation index		
Lower *NAT2* demethylation index	4.62 (1.12, 19.05)	**0.034**
Higher *NAT2* demethylation index	Reference	

*P* values marked with bold indicate statistically significant differences between the groups.

*ATDILI* anti-tuberculosis drug-induced liver injury, *CI* confidence interval, *NAT2* *N*-acetyltransferase 2, *OR* odds ratio.

^a^Adjusted for age, gender, body mass index (BMI), drinking status, smoking status, and timing of blood collection.

### Negative correlation between *NAT2* demethylation index and clinical parameters of tuberculosis patients

**Fig. 3:**
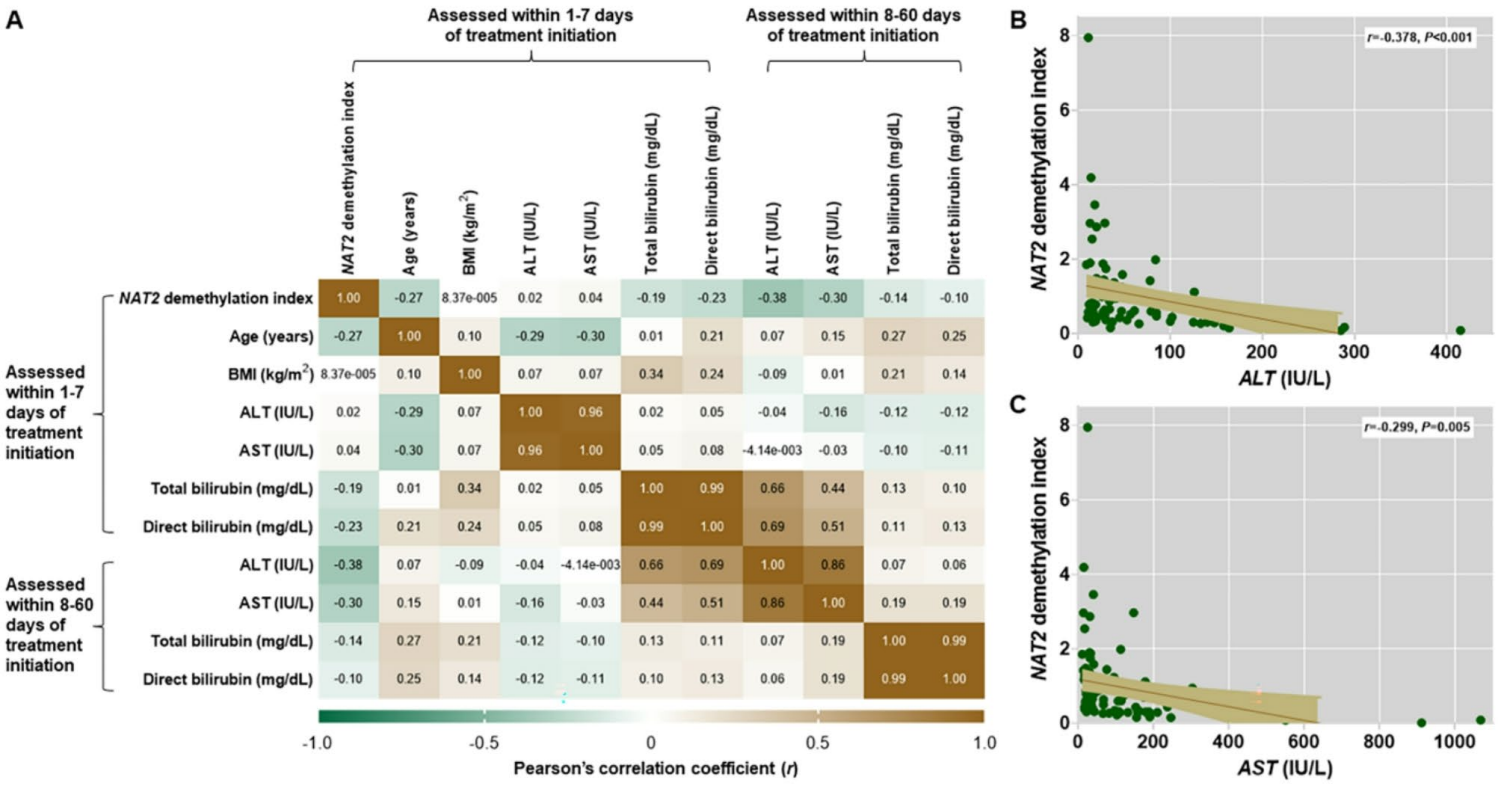
Correlation between *NAT2* demethylation index and clinical parameters indicating ATDILI progression in tuberculosis patients. (**A**) Heatmap of Pearson correlation matrix between *NAT2* demethylation index and clinical parameters of ATDILI. (**B**) Scatter plot displaying an inverse correlation between *NAT2* demethylation index measured within 1–7 days of treatment and serum ALT levels measured within 8–60 days of treatment. (**C**) Scatter plot displaying an inverse correlation between *NAT2* demethylation index measured within 1–7 days of treatment and serum AST levels measured within 8–60 days of treatment.* P*-values were derived from Pearson’s correlation.

It was further determined if *NAT2* demethylation index was correlated with clinicopathological parameters indicating ATDILI development. The correlation matrix between *NAT2* demethylation index and clinicopathological parameters assessed within either 1–7 days or 8–60 days of starting treatment in tuberculosis patients is revealed in Fig. 3A. Pearson’s correlation analysis unveiled that *NAT2* demethylation index, measured within 1–7 days of treatment initiation, was inversely correlated with serum levels of ALT and AST, assessed within 8–60 days of starting treatment, in tuberculosis patients (*r* = − 0.378, *P* < 0.001; *r* = − 0.299, *P* = 0.005; respectively) (Fig. 3B,C).

Whether *NAT2* demethylation index was independently associated with clinical parameters was further determined through multivariate linear regression analysis. After adjusting for age, gender, BMI, drinking status, smoking status, and the timing of blood collection, a decrease in *NAT2* demethylation index observed within 1–7 days of treatment was found to be independently associated with higher serum levels of ALT and AST measured 8–60 days after starting treatment in tuberculosis patients (β-coefficient = − 0.005; 95% CI − 0.007, − 0.002; *P* < 0.001; β-coefficient = − 0.002; 95% CI − 0.003, − 0.001; *P* = 0.005, respectively).

### *NAT2* demethylation index as an early biomarker for ATDILI

**Fig. 4:**
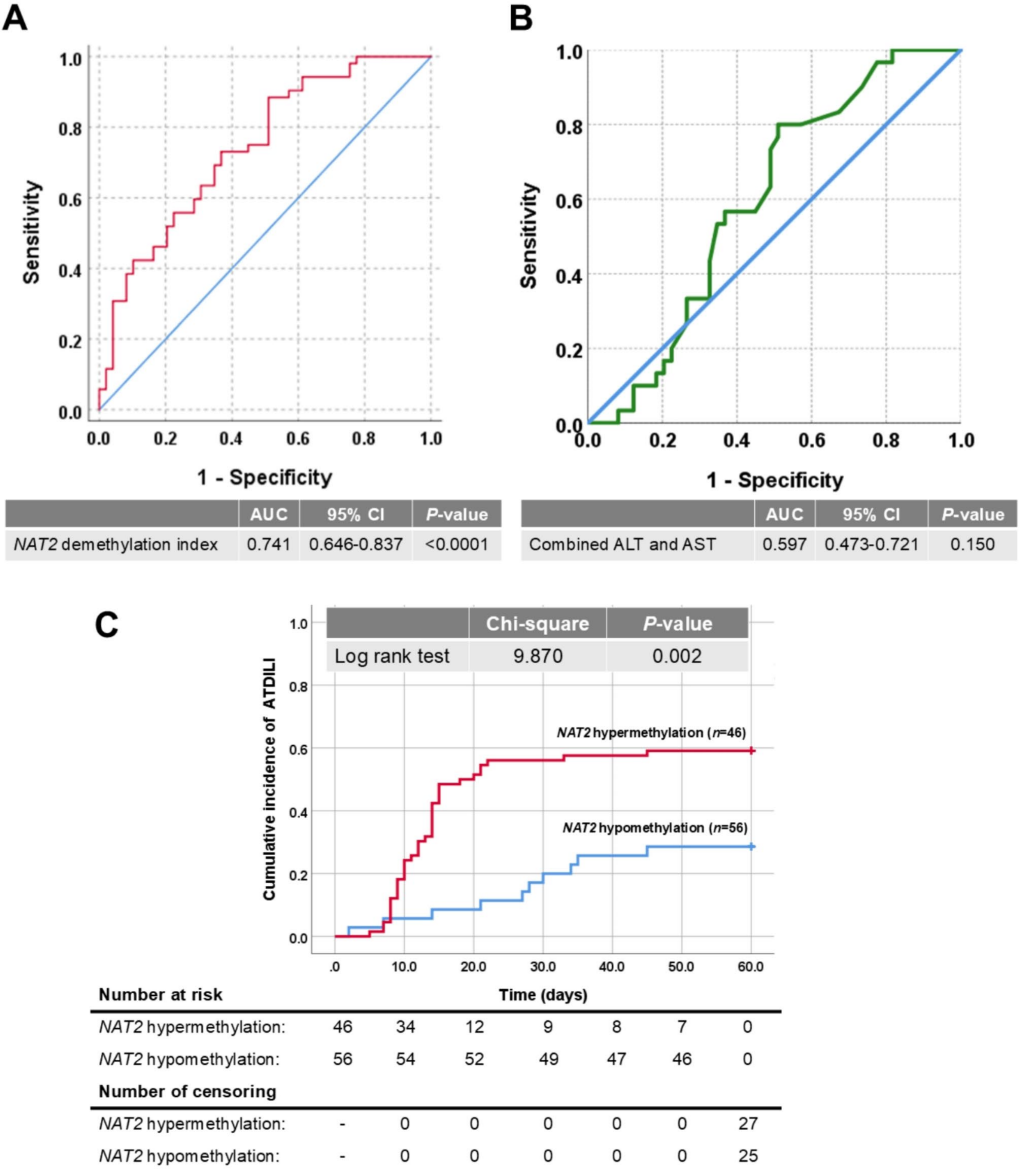
*NAT2* demethylation index measured within 1–7 days after starting tuberculosis treatment as a diagnostic and prognostic biomarker for identifying tuberculosis patients at risk of ATDILI. (**A**) ROC demonstrating the AUC of *NAT2* demethylation index. (**B**) ROC demonstrating the combined AUCs of ALT and AST. (**C**) Kaplan–Meier curve unveiling significant association between a decrease in *NAT2* demethylation index and an increased rate of ATDILI occurrence. Along the Kaplan–Meier survival curve, the censoring mark represented by a plus sign (+) indicates the time point at which participants were censored, meaning that they had not yet experienced ATDILI by the end of the study period.

To assess the potential use of *NAT2* demethylation index as an early ATDILI biomarker, the area under the ROC curve (AUC) was calculated. Following the initiation of tuberculosis treatment within 1–7 days, the detection of *NAT2* demethylation index proved to be considerably more effective in distinguishing ATDILI patients from non-ATIDLI patients in tuberculosis patients, compared to ALT and AST. For discriminating tuberculosis patients with ATDILI from those without ATDILI, ROC curve analysis revealed that *NAT2* demethylation index of 0.52 yielded a sensitivity of 73.10, a specificity of 63.30, a positive predictive value (PPV) of 61.11, a negative predictive value (NPV) of 67.86, and an AUC of 0.741 (95% CI 0.646, 0.837; *P* < 0.001) (Fig. 4A). However, the combined AUCs of ALT and AST measured within 1–7 days of treatment initiation did not demonstrate statistical significance (Fig. 4B).

### Association between *NAT2* hypermethylation and an increased rate of ATDILI occurrence

Considering lower *NAT2* demethylation index as an independent determinant of ATDILI, we proceeded to investigate its effect on the occurrence of ATDILI in tuberculosis patients. Kaplan–Meier analysis uncovered that tuberculosis patients with lower *NAT2* demethylation index had a significant higher cumulative rate of ATDILI occurrence compared to those with higher *NAT2* demethylation index (log-rank: χ^2^ = 9.870, *P* = 0.002) (Fig. 4C). After adjustments with confounders including age, gender, BMI, drinking status, smoking status, and the timing of blood collection, Cox regression analysis confirmed the above observation that lower *NAT2* demethylation index was associated with a higher rate of ATDILI occurrence in tuberculosis patients (hazard ratio, HR 2.617; 95% CI 1.107, 6.182; *P* = 0.028).

## Discussion

Despite extensive research on the role of genetic variants in hepatotoxic mechanisms^27^, these risk factors alone do not fully explain the variability in ATDILI susceptibility among individuals. The complex pathogenesis of ATDILI suggests that additional, non-genetic factors may contribute to interindividual differences in the risk of ATDILI. DNA methylation, an epigenetic mechanism regulating gene expression by integrating genetic and environmental factors, has emerged as a potential contributor to ATDILI pathogenesis. Previous studies have reported associations between differential DNA methylation levels in promoter regions of genes encoding metabolic enzymes—such as *CYP2E1, CYP2D6, GSTP1,* and *NAT2*—and ATDILI risk in tuberculosis patients^17,18,21^. Furthermore, a genome-wide DNA methylation analysis has identified significant alterations in methylation patterns linked to ATDILI progression^15^. Building upon this evidence, our study investigated *NAT2* demethylation index in blood leukocytes of tuberculosis patients with and without ATDILI and uncovered a significant decrease in *NAT2* demethylation index in tuberculosis patients with ATDILI compared to non-ATDILI patients and healthy controls. In addition to this, our findings revealed an independent association between *NAT2* demethylation index and an increased risk of ATDILI in tuberculosis patients. Notably, *NAT2* demethylation exhibited an inverse correlation with serum aminotransferase levels in tuberculosis patients within 8–60 days after initiating treatment. Although this negative correlation between *NAT2* hypermethylation and ALT/AST levels was statistically significant, its strength was relatively weak. This suggests that while *NAT2* methylation might contribute to ATDILI susceptibility, it is unlikely to be the sole factor influencing serum ALT/AST elevations. To establish a more precise temporal relationship, a longitudinal study with multiple time points assessing both *NAT2* methylation and ALT/AST levels is warranted. In support of our results, a clinical study by Zhang et al.^21^ previously shed light on *NAT2* hypermethylation in Mongolian tuberculosis patients with ATDILI. In the light of the aforementioned findings, it has been postulated that *NAT2* hypermethylation might be implicated in the development and progression of ATDILI in tuberculosis patients. Furthermore, it holds promise as a potential epigenetic biomarker for the early detection of ATDILI. Our further results from ROC curve analysis depicted that measuring *NAT2* demethylation index within 1–7 days after starting TB treatment appears to be more accurate in distinguishing tuberculosis patients with ATDILI from those without, compared to serum aminotransferases detected within the same time frame after treatment initiation. Besides this, our in-depth analysis revealed a strong relationship between a lower *NAT2* demethylation index and an increased occurrence of ATDILI in tuberculosis patients. Altogether, the combination of previous research and our own findings provided valuable insight into the usefulness of epigenetic biomarkers, particularly *NAT2* promoter methylation, in predicting and monitoring ATDILI progression. Based on our findings, the integration of *NAT2* promoter methylation analysis into clinical practice holds considerable promise, but several critical factors must be addressed. First, the use of blood samples for DNA methylation analysis is non-invasive and practical, providing a significant advantage over more invasive diagnostic approaches. In addition to this, methods, such as qMSP and pyrosequencing, offer reliable and cost-effective means for assessing methylation status, though the choice of technique will depend on the resources available within clinical settings. While qMSP is widely utilized due to its affordability and simplicity, more advanced approaches like next-generation sequencing (NGS) offer enhanced sensitivity, though their higher cost may limit routine clinical use. For *NAT2* methylation analysis to become a part of standard clinical practice, further validation through large-scale, multicenter studies is necessary to establish its predictive accuracy and determine standardized cut-off values for clinical application. Additionally, integrating methylation testing into clinical workflows will require close collaboration between clinical and molecular laboratories to ensure the timely and precise generation of results. Cost-effectiveness and accessibility, particularly in resource-limited settings, must also be addressed to facilitate broad implementation, especially in regions where tuberculosis is endemic. Ultimately, overcoming these challenges would enable *NAT2* methylation analysis to complement existing clinical biomarkers and genetic tests, offering a valuable tool for the early detection and personalized management of ATDILI risk in tuberculosis patients.

In lights of our considerations, although the precise mechanism behind *NAT2* hypermethylation in ATDILI remains incompletely comprehended, it is possible that *NAT2* hypermethylation might be caused by the buildup of reactive oxygen species (ROS), which in turn could lead to the persistent production of oxidative stress. It is well-known that ROS can induce DNA damage and influence gene expression through epigenetic modifications, including DNA methylation. Several studies have demonstrated that oxidative stress can alter DNA methylation patterns, thereby contributing to gene silencing, a process that could extend to *NAT2* as well^28,29^. While these studies do not directly address *NAT2*, they provide a scientific foundation for the potential link between ROS-induced DNA methylation and the regulation of gene expression^30^. Notably, an experimental study has shown that anti-tuberculosis drugs can potentially cause hepatocellular damage in mice through an increase in ROS production^31^. This body of research indirectly supports the hypothesis that ROS-induced methylation changes could be relevant in *NAT2* methylation in the context of ATDILI. Apart from these, abnormal DNA methylation is widely acknowledged as a common factor that can impede gene expression, aside from gene deletion and point mutation^32^. From this, hypermethylation in the promoter region of *NAT2* can result in a decrease or complete loss of *NAT2* expression, thereby altering the levels and function of NAT2 in the body. This, in turn, diminishes the acetylation metabolism of hydrazines, possibly causing an increased likelihood of liver injury^20,21,30^. Supporting the aforementioned assumption, in a study conducted by Wakefield et al.^24^, it was discovered that increased methylation in the promoter region of *NAT2* in mice can lead to the suppression of *NAT2* gene expression. This suppression can result in developmental malformations and cancer. Regarding the relationship between CpG island methylation in *NAT2* and ATDILI, a previous study unveiled that alterations in DNA methylation in the promoter region of *NAT2* might be linked to the progression of ATDILI^21^. Specifically, hypermethylation of CpG islands in the *NAT2* promoter region can typically result in gene silencing, leading to decreased *NAT2* expression and reduced enzymatic activity. This reduction in NAT2 activity might impair the detoxification of reactive drug metabolites^33^. Backing up this hypothesis, a study conducted by Lee et al.^34^ has revealed that the increased level of methylation in the promoter region of *NAT2* genes in mice inhibited *NAT2* expression, leading to the development of cancer. From this previous finding, it can be inferred that hypermethylation in the promoter region of *NAT2* gene could potentially result in reduced or even complete loss of *NAT2* expression. This, in turn, could alter the levels and activity of NAT in vivo. In addition to this, our findings of *NAT2* promoter hypermethylation in patients with ATDILI support the hypothesis that epigenetic modifications of *NAT2* might predispose individuals to hepatotoxicity during anti-tuberculosis therapy.

Although this study presents important findings, it is important to acknowledge its inherent limitations. One limitation is the inability to investigate the *NAT2* demethylation index in liver-specific cells of tuberculosis patients with ATDILI. However, it has been noted that there was a correlation between methylation levels of a specific tissue and peripheral blood^35^. Furthermore, DNA methylation profiles derived from peripheral blood leukocytes have been linked to systemic epigenetic modifications in drug response studies^36^. Based on this premise, measuring *NAT2* demethylation index in blood leukocytes might serve as a surrogate for assessing methylation changes in hepatic tissue. Nevertheless, a more comprehensive analysis of *NAT2* methylation and its corresponding mRNA expression in both liver tissue and blood leukocytes is necessary to determine the biological relevance of peripheral blood-based measurements in relation to hepatic gene expression. In addition to assessing DNA methylation in blood leukocytes, the analysis of plasma-free DNA (cfDNA), derived from apoptotic and necrotic cells, could provide a more comprehensive perspective on drug-induced epigenetic changes, including those affecting hepatocytes, the primary site of drug metabolism^37^. However, the use of cfDNA for methylation analysis presents certain technical challenges, such as low DNA yield, fragmentation, and variability in methylation patterns due to diverse cellular origins^38^. For these reasons, a comparative study investigating both peripheral blood leukocyte-derived and cfDNA methylation profiles would be valuable in determining the most reliable biomarker source for ATDILI detection. Another limitation is the insufficient data on oxidative stress, which hinders the establishment of a definitive link between *NAT2* hypermethylation and increased oxidative stress in tuberculosis patients with ATDILI. Similarly, the absence of comprehensive data on co-morbidities associated with ATDILI complicates the interpretation of our finding that *NAT2* hypermethylation was independently associated with a higher incidence of ATDILI in tuberculosis patients. Furthermore, while the study identified a strong epigenetic association, it did not establish a direct causal relationship between *NAT2* hypermethylation and ATDILI. A prospective cohort study with longitudinal data would be required to validate the predictive potential of this biomarker. Despite the observational nature of this study, the findings remain statistically robust, as evidenced by the significant differences in methylation patterns between ATDILI and non-ATDILI patients. Moreover, multivariate analyses were performed to account for potential confounding factors, further reinforcing the validity of the observed association. To enhance the reliability of these findings, future prospective studies with larger sample sizes, combined with functional validation through in vitro and in vivo models, are warrented to elucidate the mechanistic role of *NAT2* promoter methylation in ATDILI pathogenesis. Additionally, although the sample size in this study yielded valuable preliminary findings, a larger multicenter study is necessary to validate these results and improve statistical power.

In addition to NAT2, other drug metabolizing enzymes like glutathione-S-transferase (GST) and cytochrome P450 2E1 oxidase (CPY2E1) have been reportedly involved in isoniazid biotransformation^39^. From this premise, it is reasonable to speculate that alterations in the activity of GST and CPY2E1 could result in the accumulation of metabolic precursors, potentially contribute to the development of hepatotoxicity. To gain a more comprehensive understanding of the involvement of DNA methylation in ATDILI pathogenesis, it is imperative to conduct further research on methylation levels of additional genes encoding drug metabolizing enzymes, including *GST* and *CYP2E1*, alongside *NAT2*.

To sum up, this study provided groundbreaking evidence uncovering *NAT2* hypermethylation in blood leukocytes of tuberculosis patients, particularly those with ATDILI. Specifically, a significant reduction in *NAT2* demethylation index observed within 1–7 days of initiating tuberculosis treatment was found to be correlated with elevated serum aminotransferases detected within 8–60 days of starting treatment in tuberculosis patients. ROC curve analysis revealed that *NAT2* demethylation index was more sensitive and specific in distinguishing ATDILI cases from non-ATDILI cases compared to serum aminotransferases in tuberculosis patients within 1–7 days of treatment initiation. This finding was supported by the Kaplan–Meier analysis, which showed that tuberculosis patients with lower *NAT2* demethylation index had a significantly increased rate of ATDILI occurrence compared to those with higher *NAT2* demethylation index. This emphasizes the potential use of *NAT2* demethylation index as a diagnostic biomarker for ATDILI in tuberculosis patients. To establish the viability of *NAT2* demethylation index as an epigenetic biomarker for ATDILI, it is necessary to conduct a prospective cohort study for future validation.

## Electronic supplementary material

Below is the link to the electronic supplementary material.

Supplementary Material 1

## Data Availability

The data that support the findings of this study are available from the corresponding author upon reasonable request. Some data may not be made available because of privacy or ethical restrictions.
