# Supplementary material for: Association of NAT2 promoter hypermethylation with susceptibility to hepatotoxicity due to antituberculosis drugs and biomarker potential
Source: Sci Rep. 2025 Mar 25;15:10197. doi: 10.1038/s41598-025-95050-6 (PMC11937569; doi:10.1038/s41598-025-95050-6)
Supplement: Supplementary file 1 — Supplementary Material 1 [file 41598_2025_95050_MOESM1_ESM.docx]

**Supplementary information**

**Table S1** Baseline and clinical characteristics of tuberculosis patients and healthy volunteers.

| **Variables** | **Tuberculosis patients** | **Healthy volunteers** | ***P-*value^a^** |
| --- | --- | --- | --- |
| **Number** | 102 | 100 | N/A |
| **Age (years)** | 45.00 (37.00, 62.00) | 43.00 (36.00, 54.00) | 0.279 |
| **Gender (F / M)** | 37 (36.27%) / 65 (63.73%) | 40 (40.00%) / 60 (60.00%) | 0.586 |
| **BMI (kg/m^2^)** | 19.50 (16.65, 21.20) | 19.43 (16.55, 22.05) | 0.898 |
| **Drinking status** |  |  |  |
| Never / Ever | 64 (62.75%) / 38 (37.25%) | 65 (65.00%) / 35 (35.00%) | 0.739 |
| **Smoking status** |  |  |  |
| Never / Ever | 64 (62.75%) / 38 (37.25%) | 65 (65.00%) / 35 (35.00%) | 0.739 |
| **Biochemical parameters** |  |  |  |
| ALT (IU/L) | 25.00 (12.00, 35.00) | 25.00 (23.00, 30.00) | 0.720 |
| AST (IU/L) | 22.00 (15.00, 28.00) | 24.00 (15.00, 27.50) | 0.355 |
| Total bilirubin (mg/dL) | 0.80 (0.50, 1.15) | 0.50 (0.30, 0.75) | 0.407 |
| Direct bilirubin (mg/dL) | 0.30 (0.18, 0.50) | 0.22 (0.13, 0.28) | 0.372 |

Data are represented as either median with interquartile ranges (IQR) for continuous variables or percentages for categorical variables.

*P*-values marked with bold indicate statistically significant differences between the groups.

^a^Comparisons in baseline demographic and clinical parameters between tuberculosis patients with ATDILI and those with non-ATDILI.

Abbreviations: ALP, alkaline phosphatase; ALT, alanine aminotransferase; AST, aspartate aminotransferase; ATDILI, anti-tuberculosis drug-induced liver injury; BMI, body mass index; F, female; N/A, not available; M, male.
